# Elevated Pressures on Aortic Valve Leaflets correlate with Orientation of LVAD Outflow Graft

**DOI:** 10.1186/1749-8090-10-S1-A356

**Published:** 2015-12-16

**Authors:** Christof Karmonik, Mahwash Kassi, Arvind Bhimaraj, Jerry Estep, Matthias Loebe, Su Ming Chang

**Affiliations:** 1 Houston Methodist Hospital, 6565 Fannin Street, Houston, TX 77030, USA

## Background/Introduction

Better understanding of hemodynamic alterations caused by long-term use of continuous-flow left ventricular assist devices (LVAD) is necessary to evaluate their impact onto the native heart and major vessels. In particular, aortic insufficiency is a known complication after LVAD implantation and may be related to alteration of pressure distribution on the leaflets of the aortic valve.

## Aims/Objectives

To evaluate whether orientation of LVAD outflow graft orientation correlates with altered hemodynamics at the aortic leaflets.

## Method

Computational fluid dynamics (CFD) simulations were performed in models derived from cardiac-gated computed tomographic angiography (CTA), images visualizing the leaflets of the aortic valve of seven LVAD patients. Orientation of LVAD outflow graft relative to aortic wall was related to presence of retrograde velocity inferior to the anastomosis site and to increased pressure on the aortic valve leaflets.

## Results

In four cases, the angle of the LVAD outflow graft was larger than 90 degrees (102 - 112) relative to the anterior wall of the ascending aorta inferior to the anastomosis point (i.e. 'pointing down') resulting in retrograde flow along the posterior wall towards the aortic root. Corresponding leaflets exhibited increased pressure relative to unaffected leaflets in these cases. In three cases, the LVAD outflow graft angle was smaller than 90 degrees (70 - 71, i.e. 'pointing up'). No retrograde flow or velocities were observed for these cases and no elevated pressures were observed at the aortic roots or the valve leaflets. Square root of maximum pressure on the aortic valve leaflets correlated significantly with LVAD outflow angle, correlation coefficient 0.88, p < 0.05).

## Discussion/Conclusion

Orientation of the LVAD outflow graft relative to the aortic wall, i.e. 'pointing down' or 'pointing up', has direct impact on the pressure distribution at the aortic valve leaflets with adverse conditions for the first orientation potentially promoting aortic insufficiency.

